# State Registration and Its Values

**Published:** 1920-09-18

**Authors:** 


### THE MATRONS' AND SISTERS' DEPARTMENT.

### STATE REGISTRATION AND ITS VALUES.

### IV. The Effect of the War in Uniting the Nursing Profession.

It has been shown that the State registration of nurses was inevitably retarded, first by the admitted drawbacks to any system of voluntary registration; secondly, by the undeveloped stage of organisation through which the infant profession was passing. Without unity of aims the profession could not be aided and might easily have been hindered by registration.

When war broke out an entirely new state of nursing affairs made itself felt. Within a few months the call for nurses was so incessant and acute that almost the entire profession was focussed as it were at one point. Nurses of every age and every degree of efficiency poured in a steady stream to offer their credentials at the portals hastily but soundly constructed to meet the emergency. There resulted something so strongly resembling universal registration that it is improbable so great and rapid an enumeration of the qualifications of British nurses will be seen again within this generation. The indifference and ignorance of nurses as a body about the things which belong to their profession have proved immoveable. But in these months and years of stress they were not indifferent to the call of the country. Although from the first the authorities held to a definite standard for nurses selected to bear the strain of foreign service, the need for nurses in military and Red Cross establishments was so urgent that work could be found for a vast number of women whose qualifications were in some particular irregular, as, for instance, in the size or nature of their training-school or the length of time covered by their training. In this way the authorities obtained a broad survey of the entire profession. They could gauge the number of the thoroughly qualified. They could gauge the deficiencies of second-class nurses. From this comprehensive view they were able to form conclusions.

The first result of this skilled sifting process undertaken by women with minds capable of realising the significance of the work they were carrying out was the founding of the College of Nursing. The second was the attainment of a State Register for nurses.

The order in which these two benefits were obtained is very important. The first aim in the creative minds of those who directed and distributed the nursing services during war was to secure unity in the profession. To that end there must be association, not the kind of association which stirs up discord, but the practical professional association which flourishes among people doing the same kind of work and inspired by a common purpose. The points to be determined were: Was such association possible among nurses? And, if so, was the season of war the moment to choose for attempting it?

There are some things which can be done as it were only at the point of the sword, and the unification of the nursing profession would seem to be one of them. Strongly possessed with a sense of the dangers which threatened a disorganised profession the moment war was over, the leading spirits, under the eager encouragement and support of Sir Arthur Stanley, decided for immediate action. They put forward a modest manifesto, they invited co-operation, they succeeded almost beyond expectation, and the unity of the profession was assured.

No sooner was the profession drawn together, no sooner was a feeling of unity established, than the question of State registration became at once all-important. Already the overwhelming preponderance of nurses fitted for registration had been demonstrated. It was no question in 1916 of setting a ring fence round a mere clique, who had gone through a specified term of training inaccessible to the majority, and then proceeding to dilute them irretrievably with women who had no claim, except experience, to be called nurses at all. The careful scrutiny of qualifications under the pressure of war had shown that there were two main classes: (*a*) The three or four years' certificated nurses fully ripe for State registration; (*b*) a large and for the most part very useful body of women whose training had in some respects been irregular. As *bonâ fide* nurses admitted to the Register in years of grace these less well-qualified nurses would be of benefit to the public just as they had been to the wounded. Their admittance to the Register under proper precautions could be an injury to none.

It was thus that the war brought about the unification of the nursing profession and the spontaneous movement in favour of State registration. That the latter could not usefully have been introduced prior to the former has been clearly demonstrated.

